# Supplementary material for: Cardiac Hypertrophy Changes Compartmentation of cAMP in Non-Raft Membrane Microdomains
Source: Cells. 2021 Mar 3;10(3):535. doi: 10.3390/cells10030535 (PMC8001844; doi:10.3390/cells10030535)
Supplement: Supplementary file 1 [file cells-10-00535-s001.pdf]

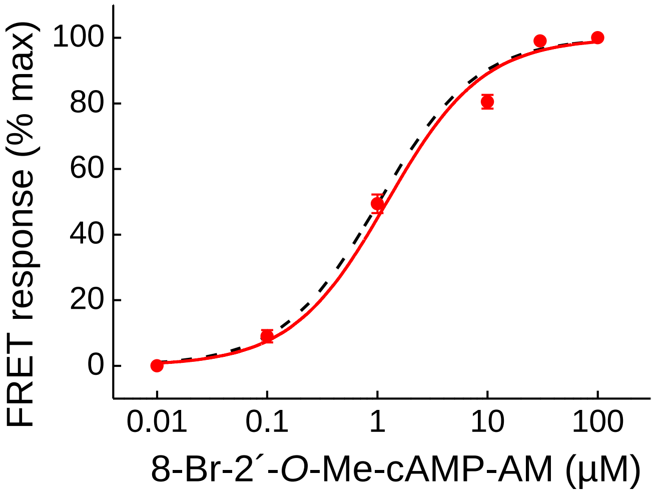

Figure S1. E1-CAAX biosensor sensitivity. Myocytes were first treated with 100 µmol/L of the adenylyl cyclase inhibitor MDL-12330A followed by increasing concentrations of the cell-permeable cAMP analogue 8-pCPT-2'-O-Me-cAMP-AM to measure biosensor affinity as described in Materials and Methods. MDL-12330A induced a substantial decrease of cAMP as evidenced by the change of CFP/YFP ratio of -11.6±0.8% as compared to basal ratio (means±SE, n=20). Concentration response dependency subsequently measured for E1-CAAX sensor with 8-pCPT-2'-O-Me-cAMP-AM (red, means±SE, n=20) is almost superimposable with that for the parental cytosolic Epac1-camps sensor established before (black dotted line, from ref. 20). EC_{50} values were 1.2±0.2 µM (E1-CAAX) and 1.2±0.1 µM (Epac1-camps).

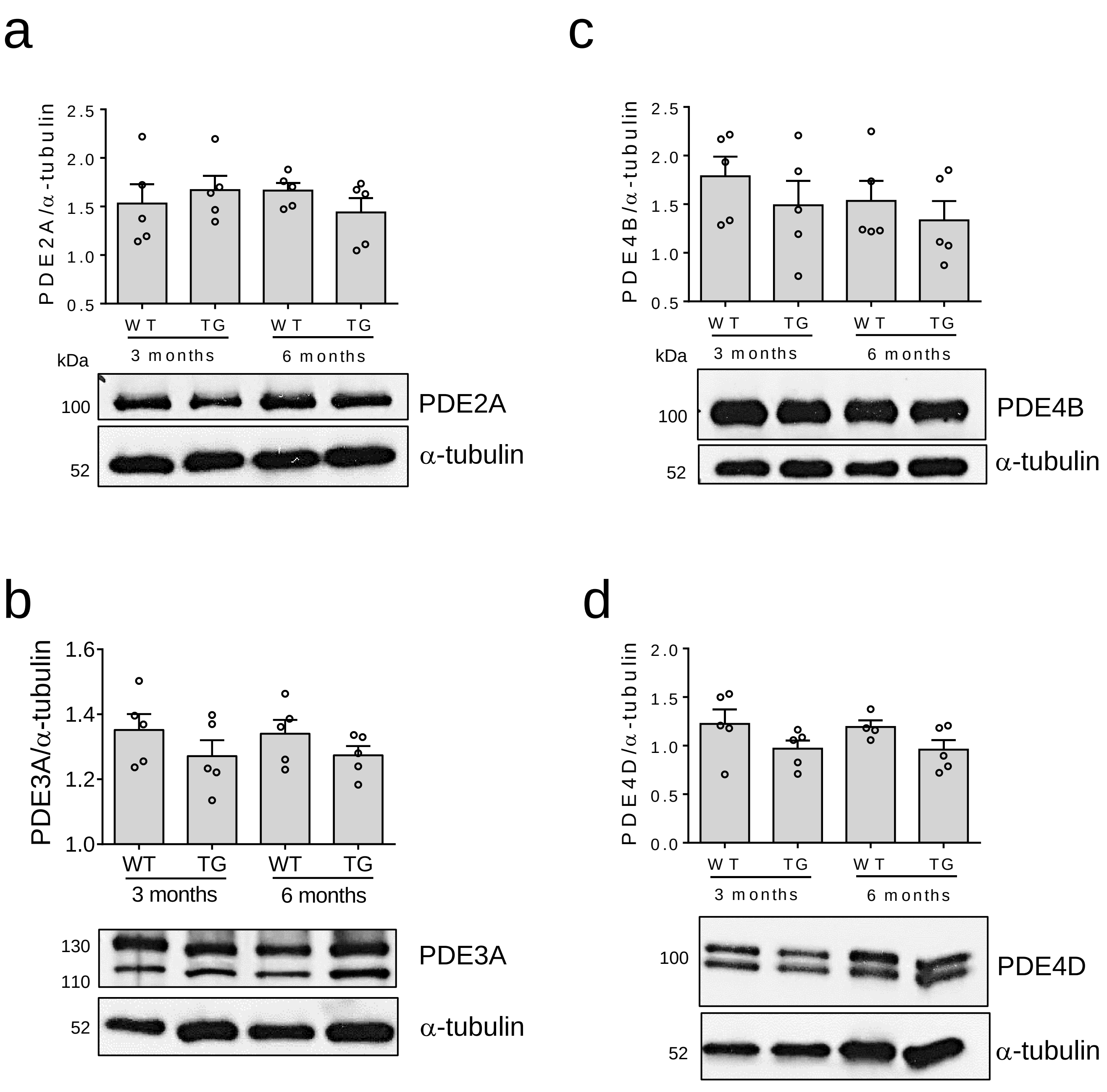

Figure S2. Expression of the major cAMP phosphodiesterases in E1-CAAX transgenic hearts (TG) as compared to hearts of wildtype (WT) littermates. Representative immunoblots for heart tissue lysates performed for 3 and 6 month old TG and WT hearts, and quantification of (**a**) PDE2A, (**b**) PDE3A, (**c**) PDE4B and (**d**) PDE4D protein expression. Means±SE.

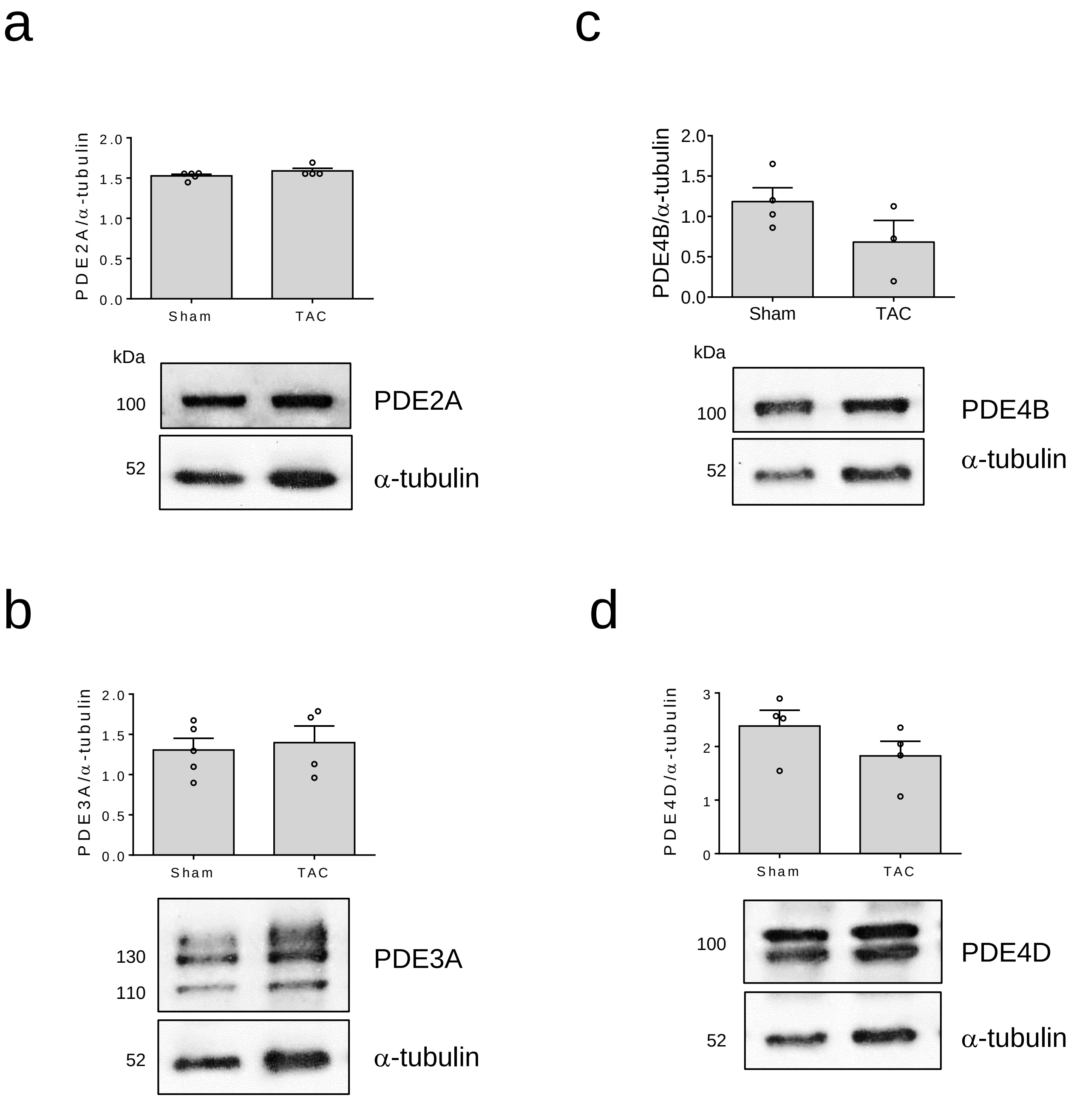

Figure S3. Expression of the major cAMP phosphodiesterases in E1-CAAX transgenic hearts 8 weeks after Sham or TAC surgery. Representative immunoblots and quantification of (**a**) PDE2A, (**b**) PDE3A, (**c**) PDE4B and (**d**) PDE4D protein expression. Means±SE.

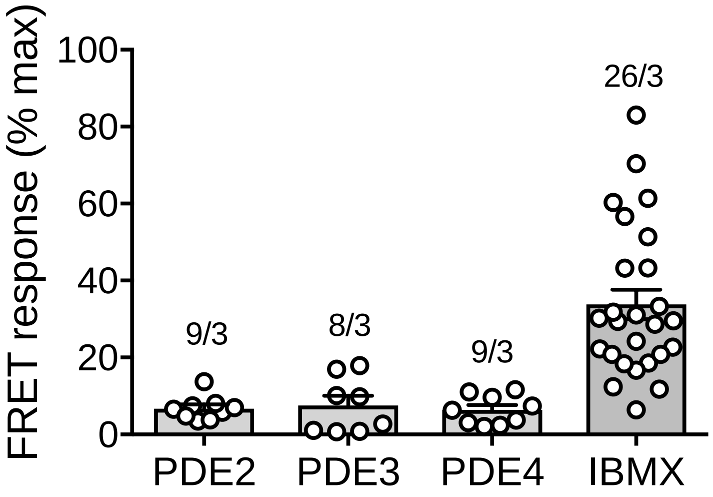

Figure S4. Effects of selective PDE inhibitors applied alone. E1-CAAX myocytes were first treated with 100 nmol/L BAY60-7550 (PDE2), 10 μmol/L cilostamide (PDE3) or 10 μmol/L rolipram (PDE4) followed by 100 μmol/L IBMX and 10 μmol/L forskolin to induced maximal biosensor response. Effects of individual PDE inhibitors were calculated as % change of CFP/YFP ratio and normalized on maximal response. Data are means±SE, number of measured cells/mice is above the bars.

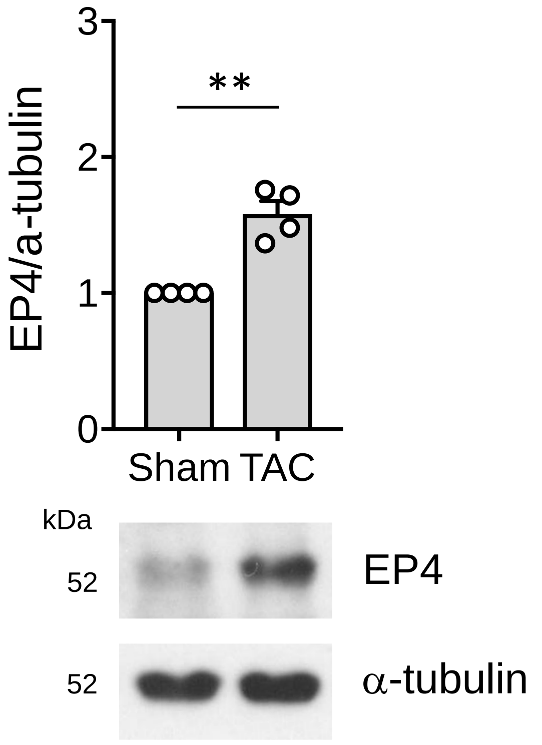

Figure S5. Expression of the EP4 prostaglandin receptor in FVB/N mouse hearts 8 weeks after Sham or TAC surgery. Representative immunoblots and quantification (n=4 hearts each). Shown are means±SE. ** significant difference at p<0.01 by one-way ANOVA.

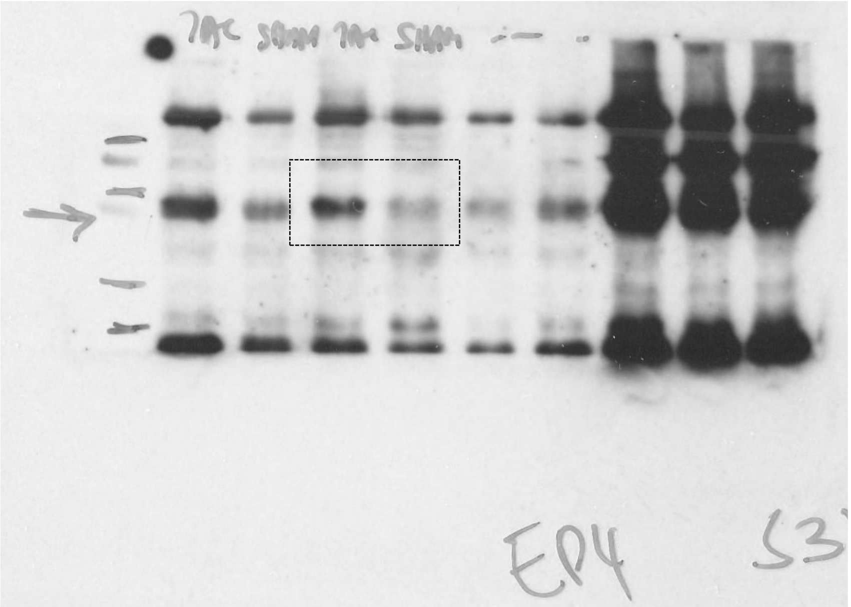

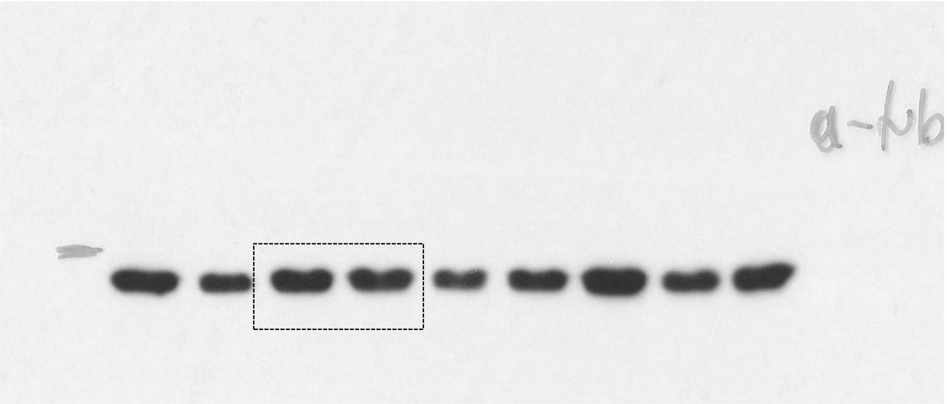

Uncropped blots for Figure S5. Expression of the EP4 prostaglandin receptor in FVB/N mouse hearts 8 weeks after Sham or TAC surgery
